# A novel miRNA analysis framework to analyze differential biological networks

**DOI:** 10.1038/s41598-017-14973-x

**Published:** 2017-11-03

**Authors:** Ankush Bansal, Tiratha Raj Singh, Rajinder Singh Chauhan

**Affiliations:** 1 Department of Biotechnology and Bioinformatics, Jaypee University of Information Technology, Waknaghat-, 173234 Solan, H.P. India; 2 Department of Biotechnology, Bennett University- A Times Group Initiative, TechZone II, Greater Noida, 201310 Uttar Pradesh India

## Abstract

For understanding complex biological systems, a systems biology approach, involving both the top-down and bottom-up analyses, is often required. Numerous system components and their connections are best characterised as networks, which are primarily represented as graphs, with several nodes connected at multiple edges. Inefficient network visualisation is a common problem related to transcriptomic and genomic datasets. In this article, we demonstrate an miRNA analysis framework with the help of *Jatropha curcas* healthy and disease transcriptome datasets, functioning as a pipeline derived from the graph theory universe, and discuss how the network theory, along with gene ontology (GO) analysis, can be used to infer biological properties and other important features of a network. Network profiling, combined with GO, correlation, and co-expression analyses, can aid in efficiently understanding the biological significance of pathways, networks, as well as a studied system. The proposed framework may help experimental and computational biologists to analyse their own data and infer meaningful biological information.

## Introduction

Complex networks theory plays a vital role in various disciplines, ranging from computer science, sociology, engineering, and physics to molecular and population biology. In the fields of biology and medicine, a network analysis may be applied for identifying a drug target, determining protein or gene function, designing effective strategies for treating various diseases, or diagnosing disorders early. Many different interaction networks, including gene regulatory interactions, transcriptional regulatory networks, and metabolic networks, emerge from the sum of transcriptomic interactions^1–3^. Network biology aims at inspecting molecular components to deduce meaningful information from large transcriptomic datasets. Generally, all metabolic networks depend on each other and form a ‘network of networks’, which is responsible for the behaviour of the biological system^4,5^. A major challenge in modern biology is to embark on an integrated theoretical and experimental programme to retrieve, comprehend, and model the parametric terms for the topological and dynamic properties of various networks that control the behaviour of the biological system, ultimately resulting in phenotypic changes.

miRNA are small regulatory noncoding molecules (approximately 22 nucleotides) that control the expression of genes at the transcript level. Gene expression alteration has a substantial effect on their respective mRNA targets and subsequently on the associated metabolic pathways^6^. miRNAs can exert an effect on most of the normal biological processes (BPs), such as immunity, metabolism, and development. Subsequently, alterations in miRNA targets cause disturbances in the molecular interaction and expression of genes. Recent studies have reported that miRNAs play crucial roles in host–gut microbiota interactions^7^, host–parasite interactions^8^, and transgenerational epigenetic inheritance^9^. Although various studies have identified correlations of several miRNAs with differential conditions on the basis of homology searches^10^, the molecular mechanism connecting miRNA and associated targets still remains unclear.

The identification of miRNA targets forms the main step towards better understanding miRNA functions^11,12^. Computational predictions have been proved to be very useful; however, they produce high false-positive rates^13^. During the past decade, high-throughput RNA sequencing has been considered a useful tool, which provided new insights into gene identification in many plant species^14,15^. For instance, RNA-seq and microarray experiments have been increasingly employed to determine the silencing effects of miRNAs at the gene expression level. The RNA-seq–based transcriptome scrutiny fastens the process of understanding plant systems in terms of biotic or abiotic stress, including virus infection, at the molecular level^16^. The mechanism of miRNA interaction between a virus and host plant was reported for various plant species including Jatropha^17^. The next-generation sequencing approaches substantially increase the quantity and quality of data on miRNA targets and their functional annotations.

Many online databases have been developed to handle emergent datasets, such as miRTarBase^18^, TarBase^19^, miRecords^20^, and starBase^21^. In the intervening time, an array of miRNA studies have generated a high quantity of data that associate miRNAs for epigenetic modifications, diseases, drug effects, and additional molecules with databases such asEpimiR^22^, Pharmaco-miR^23^, miR2Disease^24^, SM2miR^25^, and PhenomiR^26^. Mutually, these databases provide rich information to comprehend targets and potential functions for many specified miRNAs of interest. However, the target analysis and miRNA target network co-expression studies need to be deeply explored to understand associated partners and respective outcomes of gene regulatory interactions.

Even after determining targets for a specific miRNA, the identification of its functions is also a crucial task because each miRNA has the potential to target multiple different genes and consequently affect numerous BPs. To tackle this issue, scientists usually look for functions or pathways on which these miRNAs converge. A universal approach is to use enrichment analysis techniques for examining whether a given biological function unit is more frequently observed compared with the anticipation by random chance. Nonetheless, these enrichment techniques have been employed under the supposition that genes are selected consistently at random from a finite population referred as the gene universe, which may not be true in the case of target genes selected on the basis of query miRNAs.

Compared with protein-coding genes, most miRNA knockouts have very modest and subtle phenotypic effects^27^. One logical explanation is that multiple miRNAs may regulate their target genes cooperatively through a combinatorial or synergistic association. Therefore, examining the combined functions of target genes from a list of miRNAs showing synchronised changes appears to be more biologically significant. To get acquainted with such complex ‘many-to-many’ relationships between miRNAs and target genes, the best method is to use network visualisation methods. This approach associated with reliable enrichment analysis support can provide beneficial information that can help in gaining central insights into the miRNA regulatory machinery. The uncomplicated nature of this approach can identify major ‘*dramatis personae*’ from the network perspective by identifying those genes that are targeted by multiple miRNAs or that together regulate multiple genes of interest. However, such analysis and visualisation supports for NGS datasets are not available in current miRNA tools.

In this study, we have developed a novel miRNA analysis framework by using the transcriptomic datasets of *Jatropha curcas* L. as a sample input. This miRNA framework includes a six-step process where transcriptome data areannotated, followed by the miRNA identification and prediction of mRNA targets. Selected miRNA–mRNA interactions were considered for the construction of gene ontology (GO) inferred network, as a result of which differential expression analysis was performed using Pearson’s correlation coefficient (PCC). The analysed nodes with a significant fragments per kilobase million (FPKM) value were used in further co-expression analysis.

To validate the proposed framework for various datasets, we contemplated two differential conditions of Jatropha. Various viral, fungal, and bacterial infections reduce the global average yield of *J. curcas* by 16% yearly^28^, the majority of which results from the occurrence of *J. curcas* mosaic virus, which causes leaf curling and reduction in fruit size^17^. Therefore, we considered the virus-infected (JV) tissue as one of the differential conditions. To identify molecular and cellular processes leading to phenotypic variations, we also considered the healthy (JH) tissue. Archit *et al*. reported the transcriptomic and molecular mechanistic understanding of JH and JV conditions^26^. The present study displays a meticulous comparative network analysis of differential miRNA expressions and disease-specific unique miRNAs in the virus-infected tissue to understand pathway complexity.

## Results and Discussion

The miRNA analysis framework would broaden the horizon of the traditional miRNA target identification process and help in understanding the mechanism of action by using various network profiles. miRNA regulatory networks have numerous advantages over random networks because miRNAs are positioned upstream of gene signal transduction; therefore, alterations in miRNA expression are more sensitive and occur before changes in proteins^29^. In the present study, miRNA network analysis was performed using JH and JV transcriptome datasets from virus–host interactions to obtain regulatory nodes. The persistence of a large proportion of shared target proteins between JH and JV indicated that miRNA regulatory sub networks and viral infections are significantly interwoven in host cell networks. The overlapping of targeted genes involved in crucial cellular processes suggests that miRNAs play key roles in the process of viral infection.

miRNAs exert a substantial effect on targeted mRNA and gene expression. Variations in gene expression exert an effect on molecular pathways, which in turn affect cellular processes^30^. A total of 11 and 13 miRNAs are identified in JH and JV, respectively, of which 8 are common in both, namely miR-156, miR-157, miR-159, miR-319, miR-4995, miR-5021, miR-5658, and miR-f11908 (Table 1). To gain a deep insight into gene expressions, cellular processes, and associated pathways, controlling elements that are unique to particular conditions should be identified. Hence, we identified unique miRNAs in JH as miR-172, miR-414, and miR-529. In addition, the miR-2910, miR-2914, miR-477, miR-f11953, and miR-f12158 are identified uniquely in JV (Table 2). JH-specific miRNAs can be used as biomarkers to evaluate the resistance mechanism in healthy tissues, and JV-specific miRNAs can point out targets that are compromised during a virus attack. miR-f11908, miR-f11953, and miR-f12158 are novel miRNAs identified by the proposed miRNA analysis framework in *J. curcas*, and these miRNAs are also not experimentally validated in other plant species.

**Table 1:** miRNA targets common across healthy (JH) and diseased (JV) conditions.

S.No	miRNA	JH Target Name	JV Target Name	JH FPKM	JV FPKM	Regulation (up/down)
1.1	miR-156	choline monooxygenase [EC:1.14.15.7]	choline monooxygenase [EC:1.14.15.7]	2092.64	6282.87	↑
1.2	miR-156	—	ferrochelatase [EC:4.99.1.1]	—	114.97	
1.3	miR-156	—	histone H3	—	292.65	
1.4	miR-156	—	ketol acid reductoisomerase [EC:1.1.1.86]	—	261.3	
2.1	miR-157	choline monooxygenase [EC:1.14.15.7]	choline monooxygenase [EC:1.14.15.7]	2092.64	6282.87	↑
2.2	miR-157	—	ferrochelatase [EC:4.99.1.1]	—	114.97	
2.3	miR-157	—	ketol acid reductoisomerase [EC:1.1.1.86]	—	261.3	
3.1	miR-159	acetyl-CoA carboxylase, biotin carboxylase subunit [EC:6.4.1.2 6.3.4.14]	acetyl-CoA carboxylase, biotin carboxylase subunit [EC:6.4.1.2 6.3.4.14]	205.13	287.43	↑
3.2	miR-159	ubiquitin-conjugating enzyme E2 W [EC:2.3.2.25]	ubiquitin-conjugating enzyme E2 W [EC:2.3.2.25]	253.35	296.57	↑
4	miR-319	ubiquitin-conjugating enzyme E2 W [EC:2.3.2.25]	ubiquitin-conjugating enzyme E2 W [EC:2.3.2.25]	253.35	296.57	↑
5.1	miR-4995	RecQ-mediated genome instability protein 2	—	25.26	—	
5.2	miR-4995	small subunit ribosomal protein S5	small subunit ribosomal protein S5	131.65	64.02	↓
6.1	miR-5021	acetyl-CoA C-acetyltransferase [EC:2.3.1.9]	acetyl CoA C acetyltransferase [EC:2.3.1.9]	100.27	74.47	↓
6.2	miR-5021	alanine-glyoxylate transaminase/(R)-3-amino-2-methylpropionate-pyruvate transaminase [EC:2.6.1.44 2.6.1.40]	alanine glyoxylate transaminase/(R) 3 amino 2 methylpropionate pyruvate transaminase [EC:2.6.1.44 2.6.1.40]	101.03	155.47	↑
6.3	miR-5021	bud site selection protein 31	bud site selection protein 31	39.04	33.97	↓
6.4	miR-5021	DNA polymerase epsilon subunit 2 [EC:2.7.7.7]	DNA polymerase epsilon subunit 2 [EC:2.7.7.7]	32.91	35.28	↑
6.5	miR-5021	fanconi anemia group M protein	fanconi anemia group M protein	382.71	475.56	↓
6.6	miR-5021	ferulate-5-hydroxylase	ferulate-5-hydroxylase	123.23	189.44	↑
6.7	miR-5021	hydroxymethylglutaryl-CoA synthase [EC:2.3.3.10]	hydroxymethylglutaryl CoA synthase [EC:2.3.3.10]	100.27	355.36	↑
6.8	miR-5021	mRNA export factor	mRNA export factor	265.6	233.86	↓
6.9	miR-5021	nucleolar protein 58	nucleolar protein 58	88.02	151.55	↑
6.10	miR-5021	protein disulfide-isomerase A6 [EC:5.3.4.1]	protein disulfide isomerase A6 [EC:5.3.4.1]	141.6	220.8	↑
6.11	miR-5021	translation initiation factor 5B	translation initiation factor 5B	991.98	2164.84	↑
6.12	miR-5021	—	(+) abscisic acid 8′ hydroxylase [EC:1.14.13.93]	—	84.92	
6.13	miR-5021	—	1 deoxy D xylulose 5 phosphate synthase [EC:2.2.1.7]	—	154.16	
6.14	miR-5021	—	beta fructofuranosidase [EC:3.2.1.26]	—	148.94	
6.15	miR-5021	—	crossover junction endonuclease EME1	—	220.8	
6.16	miR-5021	—	glutathione reductase (NADPH) [EC:1.8.1.7]	—	265.22	
6.17	miR-5021	—	large subunit ribosomal protein L17	—	33.97	
6.18	miR-5021	—	peroxidase [EC:1.11.1.7]	—	90.15	
6.19	miR-5021	—	phosphoenolpyruvate carboxylase [EC:4.1.1.31]	—	1085.69	
6.20	miR-5021	—	photosystem I subunit X	—	57.49	
6.21	miR-5021	—	Ras GTPase activating protein 4	—	151.55	
6.22	miR-5021	—	signal recognition particle subunit SRP14	—	52.26	
6.23	miR-5021	—	small ubiquitin related modifier	—	37.89	
6.24	miR-5021	—	STIP1 homology and U box containing protein 1 [EC:2.3.2.27]	—	53.57	
6.25	miR-5021	—	tRNA specific 2 thiouridylase	—	163.31	
6.26	miR-5021	—	ubiquinone biosynthesis monooxygenase Coq6	—	145.02	
7.1	miR-5658	1-phosphatidylinositol-3-phosphate 5-kinase [EC:2.7.1.150]	1-phosphatidylinositol-3-phosphate 5-kinase [EC:2.7.1.150]	269.43	283.51	↑
7.2	miR-5658	bloom syndrome protein [EC:3.6.4.12]	—	114.05	—	
7.3	miR-5658	diacylglycerol kinase (ATP) [EC:2.7.1.107]	diacylglycerol kinase (ATP) [EC:2.7.1.107]	41.33	32.66	↓
7.4	miR-5658	DNA (cytosine-5)-methyltransferase 1 [EC:2.1.1.37]	DNA (cytosine-5)-methyltransferase 1 [EC:2.1.1.37]	229.62	320.09	↑
7.5	miR-5658	large subunit ribosomal protein L9	—	45.16	—	
7.6	miR-5658	serine/threonine-protein kinase CTR1 [EC:2.7.11.1]	serine/threonine-protein kinase CTR1 [EC:2.7.11.1]	166.09	271.75	↑
7.7	miR-5658	small subunit ribosomal protein S6	small subunit ribosomal protein S6	143.13	142.41	↓
7.8	miR-5658	transcription initiation factor TFIIF subunit alpha	transcription initiation factor TFIIF subunit alpha	244.17	282.2	↑
7.9	miR-5658	transcription-repair coupling factor (superfamily II helicase)	transcription-repair coupling factor (superfamily II helicase)	433.22	257.38	↓
7.10	miR-5658	translation initiation factor 5B	translation initiation factor 5B	991.98	2164.84	↑
7.11	miR-5658	U4/U6.U5 tri-snRNP-associated protein 2	U4/U6.U5 tri-snRNP-associated protein 2	241.11	244.31	↑
7.12	miR-5658	—	non lysosomal glucosylceramidase [EC:3.2.1.45]	—	310.94	
7.13	miR-5658	—	peptidyl prolyl cis trans isomerase like 2 [EC:5.2.1.8]	—	121.5	
7.14	miR-5658	—	RIO kinase 1 [EC:2.7.11.1]	—	299.18	
7.15	miR-5658	—	serine/threonine protein phosphatase PP1 catalytic subunit [EC:3.1.3.16]	—	84.92	
7.16	miR-5658	—	translation initiation factor eIF 2B subunit beta	—	180.29	
7.17	miR-5658	—	xanthine dehydrogenase/oxidase [EC:1.17.1.4 1.17.3.2]	—	326.62	
8.1	miR-f11908	lupus La protein	lupus La protein	120.17	94.07	↓
8.2	miR-f11908	splicing factor, arginine/serine-rich 4/5/6	splicing factor, arginine/serine rich 4/5/6	96.44	91.45	↑
8.3	miR-f11908	—	hydroxymethylpyrimidine kinase/phosphomethylpyrimidine kinase/thiamine phosphate diphosphorylase [EC:2.7.1.49 2.7.4.7–2.5.1.3]	—	134.57

**Table 2:** miRNA targets unique to healthy (JH) and diseased (JV) conditions.

S.No.	miRNA	Unique miRNA Target	Condition	FPKM
1.1	miR-172	DnaJ homolog subfamily A member 2	JH	83.43
1.2	miR-172	polyadenylate-binding protein	JH	133.95
1.3	miR-172	peroxin-5	JH	188.29
2.1	miR-414	ditrans,polycis-polyprenyl diphosphate synthase [EC:2.5.1.87]	JH	13.78
2.2	miR-414	DnaJ homolog subfamily C member 3	JH	53.58
2.3	miR-414	glycerol-3-phosphate acyltransferase [EC:2.3.1.15]	JH	55.11
2.4	miR-414	heat shock 70 kDa protein 1/8	JH	50.52
3.1	miR-529	1,4-alpha-glucan branching enzyme [EC:2.4.1.18]	JH	578.65
3.2	miR-529	phosphatidylinositol phospholipase C, delta [EC:3.1.4.11]	JH	188.29
4.1	miR-2910	4 coumarate CoA ligase [EC:6.2.1.12]	JV	100.6
4.2	miR-2910	imidazoleglycerol phosphate dehydratase [EC:4.2.1.19]	JV	103.21
5	miR-2914	Cu+ exporting ATPase [EC:3.6.3.54]	JV	69.24
6	miR-477	elongation factor 1 gamma	JV	101.91
7	miR-f11953	two component response regulator ARR B family	JV	160.7
8	miR-f12158	two component response regulator ARR B family	JV	160.7

In accordance with the aforementioned results, mRNA targets respective to the miRNAs are also identified from the transcriptome. A total of 39 and 61 targets are predicted followed by KAAS annotation in JH and JV, respectively.

**Figure 1:**
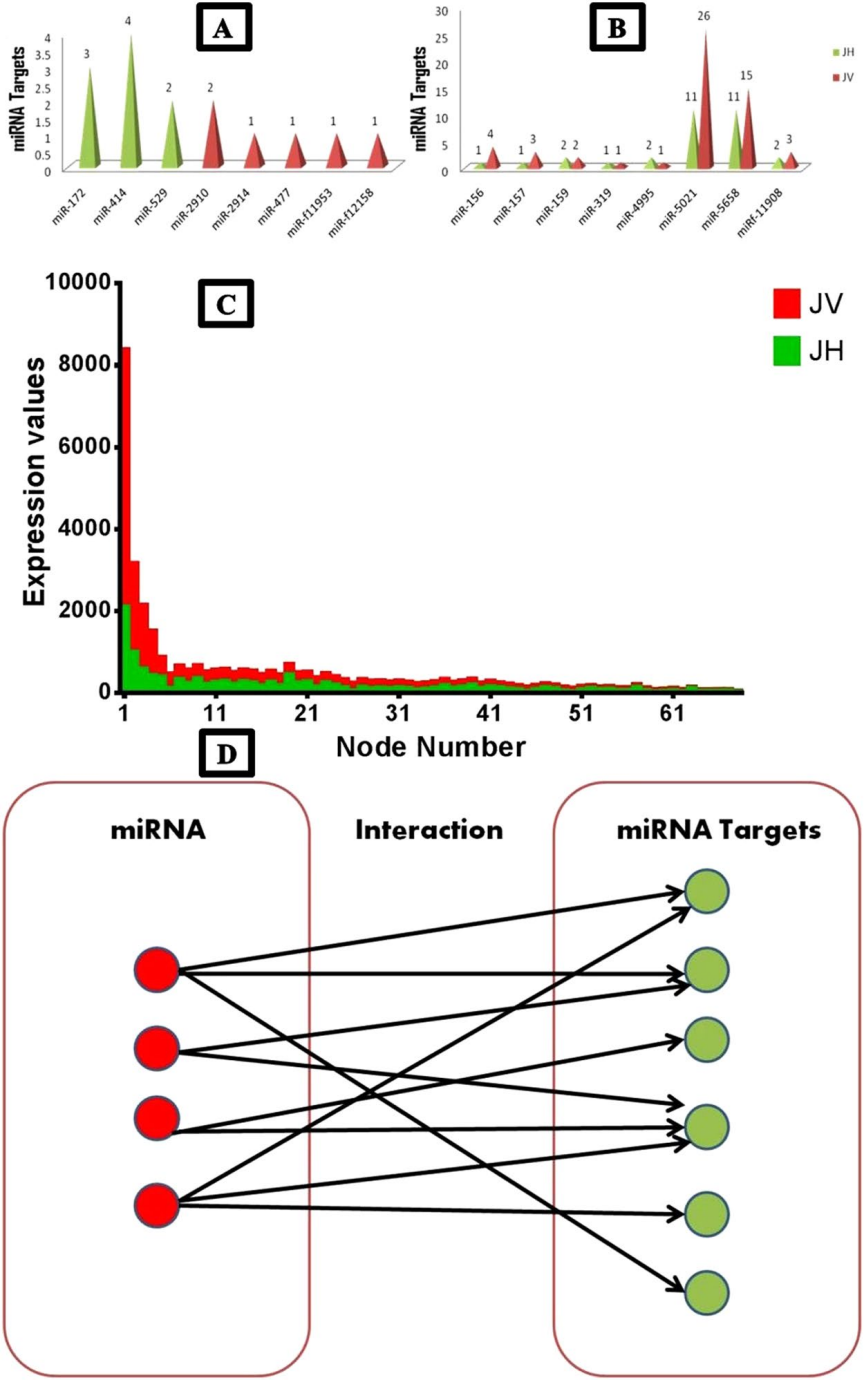
miRNA target distribution (**A**) miRNA target unique to healthy and diseased condition (**B**) miRNA targets common in both healthy and diseased conditions. miRNA targets in healthy are represented in green color while diseased in red color (**C**) Pearson Correlation Coefficient (PCC) analysis; green and red color represents healthy (JH) and diseased (JV) respectively (**D**) Bipartite network showing two different subsets, namely miRNA and miRNA target with directed connection network.

**Figure 2:**
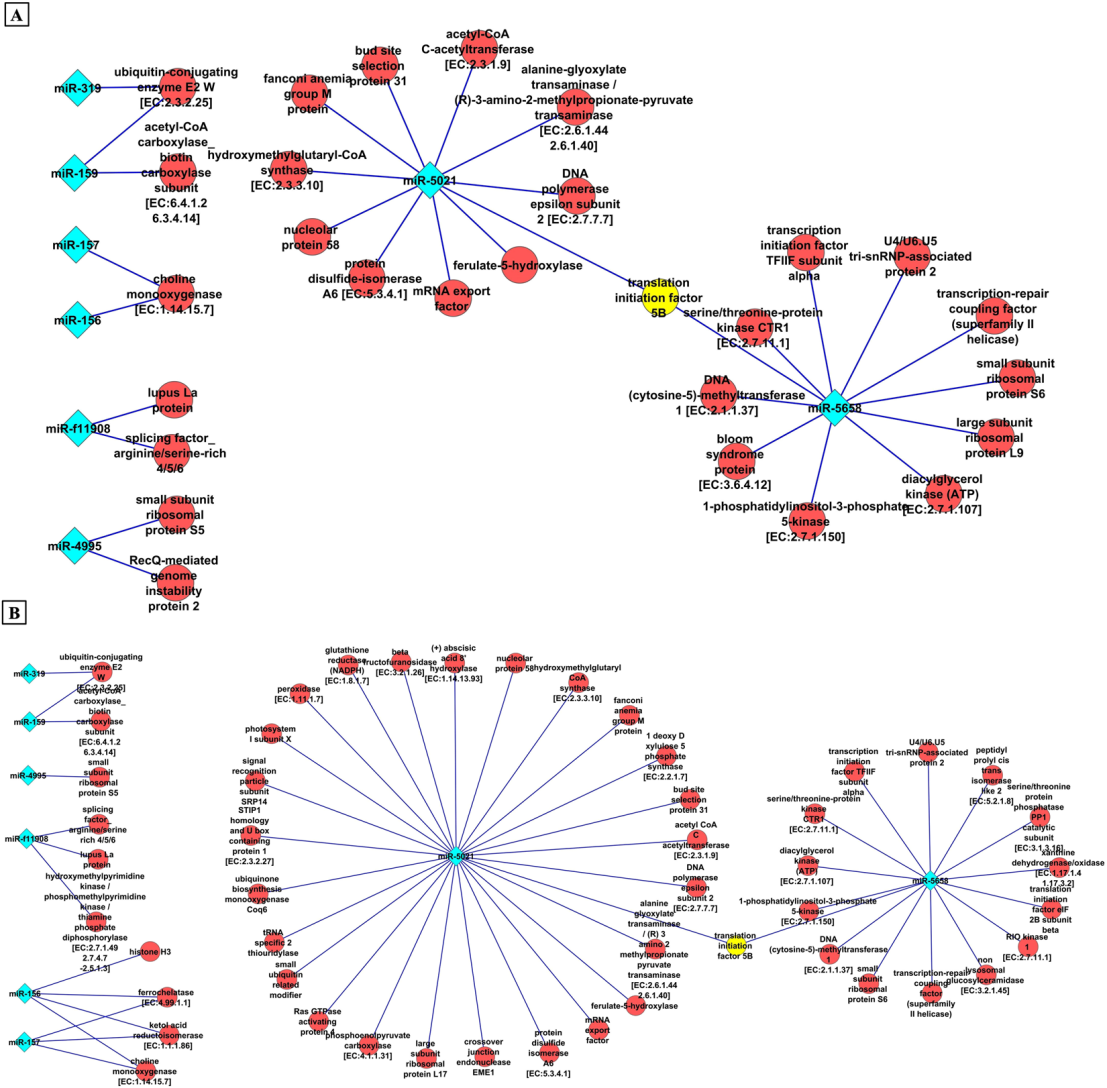
Bipartite network for common miRNA targets in (**A**) healthy (JH) condition (**B**) diseased (JV) condition.

To quantify the target transcripts of respective miRNAs in JH and JV (Fig. 1A,B), selected transcripts were presented as nodes, and their interactions were represented through edges. Only 50 and 74 interacting nodes from JH and JV, respectively, were used for further construction of the bipartite network according to miRNA–mRNA target distribution. Here, the bipartite network has been constructed to represent the association between two groups without having any connexion within the same group. On the construction of these networks, some nodes showed a dominant effect compared with other nodes (Fig. 2A,B).

Virus has an asymptotic effect on the phenotypic appearance of plants through various cellular processes controlled by various genes involved in molecular pathways^31^. Analysis of the miRNA–mRNA target network shows the major contribution of miR-5021 and miR-5658 to the regulation of the expression of various transcripts in JH and JV. Some nodes, such as choline monooxygenase, histone H3, and ferrochelatase, showed an association with more than one miRNA. To understand the BPs, molecular functions (MFs), and cellular components (CCs) of selected transcripts in JH and JV, GO analysis was performed.

GO explains a set of clearly defined, ordered vocabularies with the aim of describing the BPs, MFs, and CCs of selected transcripts. Transcripts were clustered into different subgroups and assigned a node score value, which was further used to calculate the association within clusters. Apart from BLAST2GO (which used INTERPRO and PANTHER for analysis), we separately tested our miRNA target-associated GO terms by using GORILLA and WebGestalt GSAT. The results obtained were consistent with those of BLAST2GO, and no such biased results were found.

**Figure 3:**
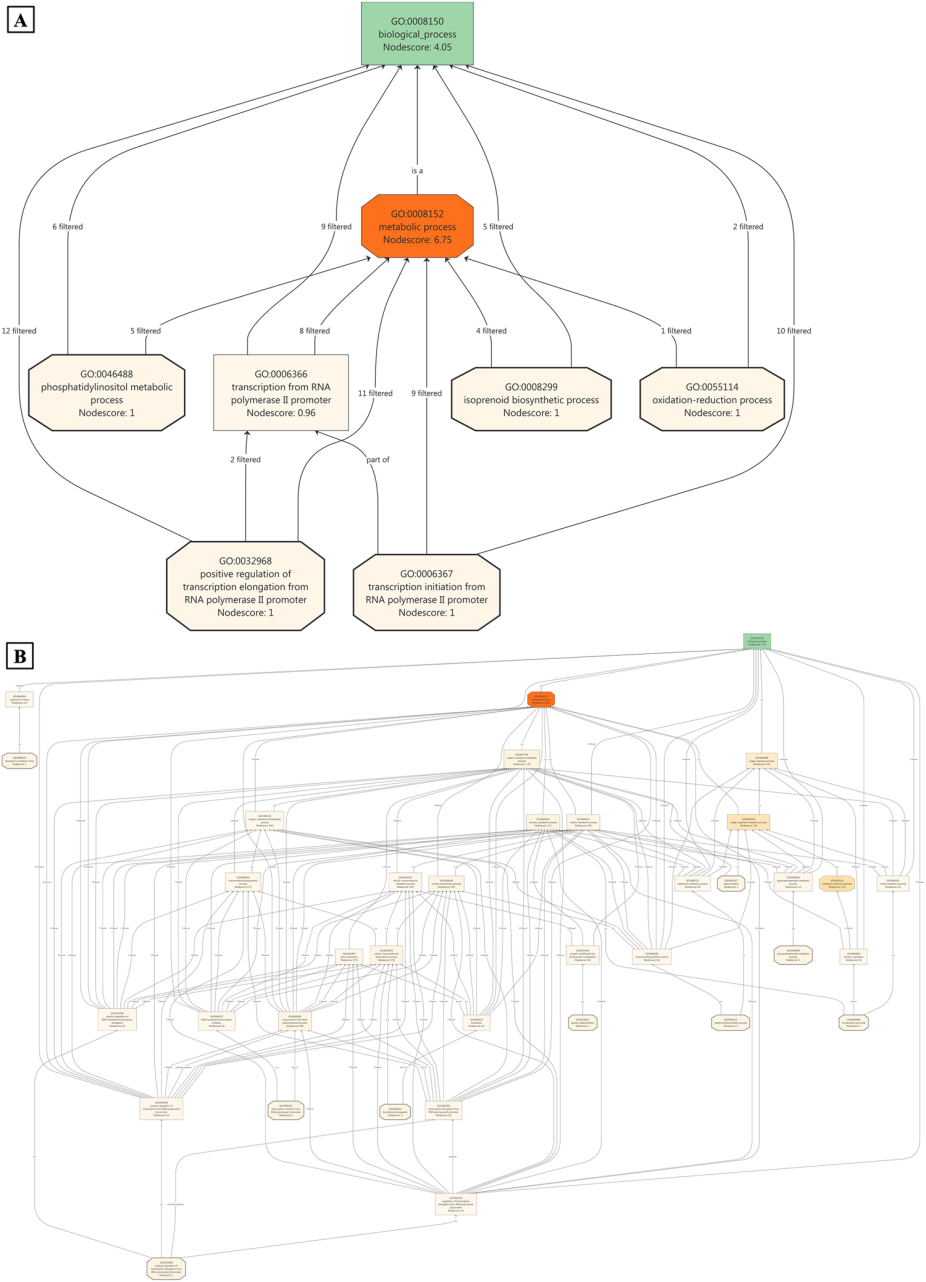
miRNA targets involved in biological processes in (**A**) healthy (JH) condition (**B**) diseased (JV) condition; score based node prioritization shown using filter parameter.

The transcripts involved in BPs in JH, such as the phosphatidylinositol metabolic process, transcription from RNA polymerase II promoter, isoprenoid biosynthesis process, oxidation reduction process, and positive regulation of the transcription elongation factor RNA polymerase II promoter, were filtered on the basis of a high GO node score (Fig. 3A). In JV, we observed more number of transcripts involved in the terpenoid biosynthesis process, aerobic respiration, tricarboxylic acid cycle, oxidative reduction process, citrate metabolic processes, organic substance biosynthetic process, and macromolecule biosynthetic processes (Fig. 3B). To cross-check and verify the role of the aforementioned BPs, we performed literature mining. According to the literature, processes involved in JH were more generalised compared with those involved in JV^32–38^. Additionally, we found that the processes mentioned in JV were involved in stress-mediated conditions to produce more energy so that plants can sustain in unfavourable conditions^39–41^.

**Figure 4:**
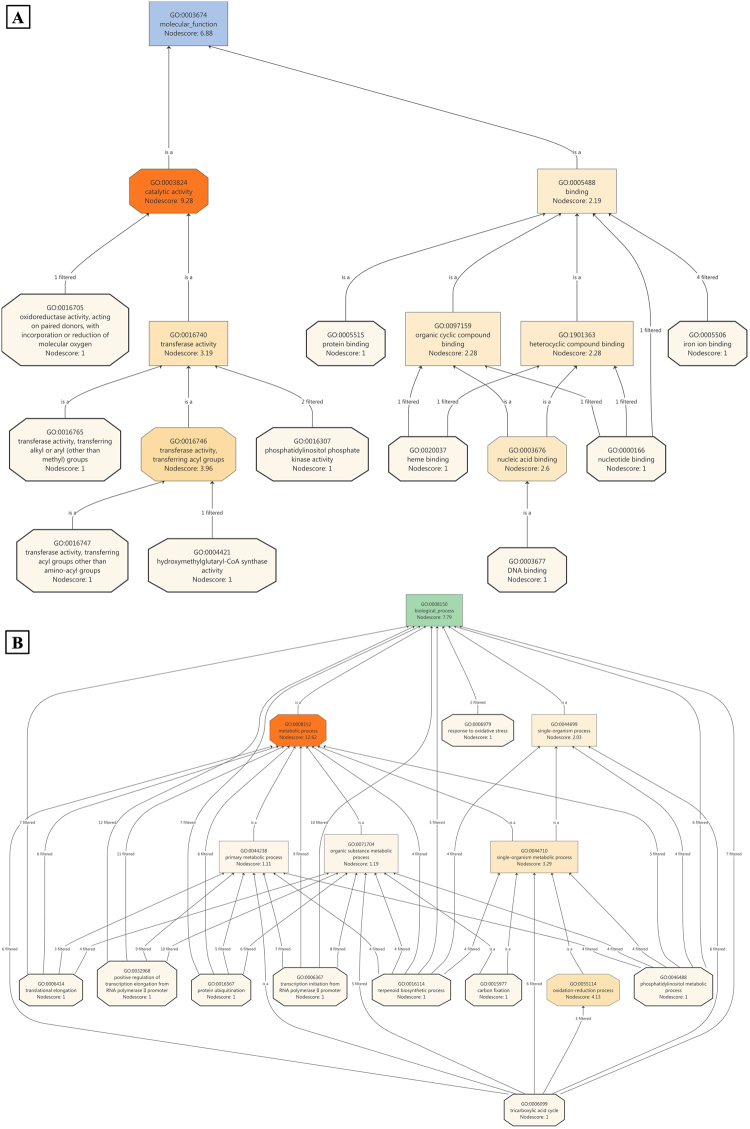
miRNA targets associated molecular functions in healthy (**A**) healthy (JH) condition (**B**) diseased (JV) condition; score based node prioritization shown using filter parameter.

**Figure 5:**
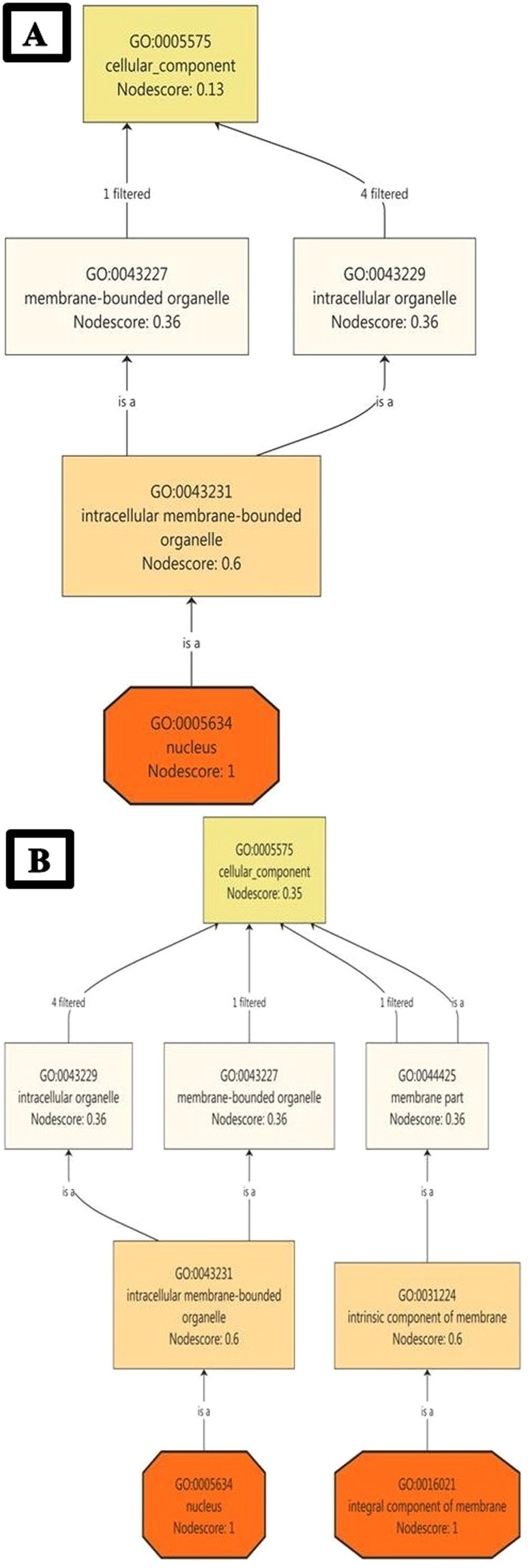
miRNA targets ontology on the basis of cellular components in (**A**) healthy (JH) condition (**B**) diseased (JV) condition; score based node prioritization shown using filter parameter.

To identify the elementary activities of genes at the molecular level, a node score was assigned to selected transcripts. Catalytic activity, transferase activity, transferase acyl activity, organic cyclic compound binding, heterocyclic binding, and nucleic acid binding were observed in JH (Fig. 4A). Response to oxidative stress, primary metabolic process, organic substance metabolic process, protein ubiquitination, transcription biosynthetic process, carbon fixation, oxidation–reduction process, and tricarboxylic acid cycle were observed in JV (Fig. 4B). A literature search of the stated MFs indicated that JH-associated functions were specific to normal conditions. However, functions relevant to JV appeared to be associated with stress and host–pathogen interactions^17,31,42^. A study of CCs in both the conditions revealed no such differential role of transcripts; however, intrinsic and integral components of the membrane were found to be uniquely present in JV (Fig. 5A,B), which indicated alteration in the cell membrane due to viral infection^43^.

PCC and Spearman’s correlation coefficient were used to evaluate the association between differentially expressed miRNA targets. However, only PCC showed a strong association within transcripts according to biological relevance. Unique miRNAs and their targets were excluded from this analysis, and only common miRNA targets were selected to observe the overall behaviour of transcripts in JH and JV conditions. As shown in Fig. 1C, miRNA target transcripts showed a significant higher expression in the JV condition than in the JH condition, and the same highly expressed miRNA target transcripts were considered for the co-expression network analysis.

The analysis of PCC results revealed10genes, namely *CMO* (miR-156 and miR-157), *RPS5* (miR-4995), *RPL9* (miR-5658), *EIF5* (miR-5658 andmiR-5021), *MVA1, PPC, PSAX, SUMO, CYP707A1*, and *DXS* (miR-5021), which were further considered inco-expression network construction (Supplementary Figs 11–12, Table 3). To further analyse the role of co-expressed genes, they were mapped with KEGG to identify pathways in which all these genes were involved^44^. *CMO*-associated co-expressed genes were found to be involved in the biosynthesis of secondary metabolites and carbon metabolism; *RPS5*-related co-expressed genes in disease resistance response; *RPL9*-related co-expressed genes in ribosomal machinery, *EIF5*-related co-expressed genes in protein processing in the endoplasmic reticulum and ubiquitin-mediated proteolysis; *MVA1*-related co-expressed genes in the biosynthesis of secondary metabolites, fatty acid biosynthesis, carbon metabolism, and fatty acid metabolism; *PPC*-related co-expressed genes in the biosynthesis of secondary metabolites, carbon metabolism, glycolysis/gluconeogenesis, and galactose metabolism; *PSAX*-related co-expressed genes in photosynthesis and photosynthesis-antenna proteins; *SUMO*-related co-expressed genes in ribosome machinery and spliceosome; *CYP707A1*-related co-expressed genes in plant hormone signal transduction; and *DXS*-related co-expressed genes in the biosynthesis of secondary metabolites, photosynthesis-antenna proteins, prohrine and chlorophyll metabolism, proteosome, and the mRNA surveillance pathway. From the results of the co-expression network construction, it can be deduced that co-expressed genes are involved in various subcellular processes, which may be controlled or altered by miRNA regulation.

**Table 3:** miRNAs, target genes, co-expressed genes and associated pathway (For more details see Supplementary File).

S. No.	miRNAs	Target Genes	Top Co-expressed genes	Co-expressed genes contributing Pathways
1	miR-156	CMO	RCC1,DUF1624,NF-YC12,MIR5344,3767731,hydrolase, kinase,tudor-like, CAMP,CHT-type C,SCAMP,MIR-834a,MBD7,TRAF-Like,NHX5,BET10,TAF6B4,DUF2358,PLDGAMMA2,TIR-NBS-LR,MRS2–7,Phosphoester	Biosynthesis of Secondary Metabolites
2	miR-157			Carbon Metabolism
3	miR-4995	RPS5	TIR-NBS-LR,ENTH,NLP7,ARM repeat, CC-NBS-LRR,PP2-A7,PP2-A6,KINASE,RFL1,F-box,Calmodulin,RPP4,RPP5,SNC1,LRR,RLM3	Disease Resistance Response
4	miR-5658	RPL9	Emb1473,RPL15,PSRP5,EMB3105,S10p/S20e,PRPL11,L19,RPS17,L28,ribosome,EMB3113, Heavy metal ion,L5P,TWN3,GHS1,ROC4,S20,emb2394,NDPK2,RPL21C	Ribosomal Machinery
		EIF5	Hydrolase,inhibitor,CYN,CUTA,NAT,819216,UBC30,GB2,G18a,GRXC2,OB-Fold Ligand,TRXH3,UBQ7,ADF6,UBC3,CHMP1A,W1H1,G8B,UBC11	Protein Processing in Endoplasmic Reticulum
5	miR-5021			Ubiquitin
		MVA1	ACP1,MOD1,PLE2,KASI,Thioesterase,BIOB,840894,CAC2,FPS1,MVD1,EMB1276,URH2,hydrolase,mutase,FaTA,NagB,UPF0041,ZHD13,CAC1-B	Biosynthesis of Secondary Metabolites
				Fatty Acid Biosynthesis
				Fatty Acid Metabolism
				Carbon Metabolism
		PPC	SOS1,MMT,alpha/beta subunit,kinase,PFK7,iPGAM2,PGM3,MDAR1,PGM2,Galactose,UGP1,mMDH2,HXK1,RR10,GLU2,JAR1,PGDH,ACO3,EMB1467,Kinase	Biosynthesis of Secondary Metabolites
				Carbon Metabolism
				Glycolysis/Gluconeogenesis
				Galactose Metabolism
		PSAX	PDAD-2,PSII,Photosynthesis,NdhS,LHCA1,PSAH-1,PSAL,LHCA3,PSAG,YCF32,PSAF,PSBW,LHCB5,PSBX,PSAN,PSAE-1,838749,PSAD-1,PSII-Q	Photosynthesis
				Photosynthesis-Antenna Proteins
		SUMO	HMGA,HTB1,HTB2,819315,TYRDC1,alpha/beta ligand,PEL3, CYP7731,LTP6,PIP2D,hydrolase,kinase,inhibitor,DRG,PME5,PDCB2,Putative mutase,	Ribosomal Machinery
				Sliceosome
		CYP707A1	AFP1,AFP3,SAG113,AB12,RAB18-PUB19,PP2-B11,SPSA2,BETAVPE,LEA7,LEA,LEA4–5,TSPO,transporter,RD29B,phosphotriase,XERO2,FMO-GS-OX-4,ESL1	Plant Hormone Signal Transduction
		DXS	GUN5,OSA1,JAC1,821278,CH1,SIGB,PSY,CP5,815980,DUF2358,COLA4,PSII,PSAD2,LHCB6,CRD1,oxidoreductase,HEMA1,818819,SLP1,rosamann	Biosynthesis of Secondary Metabolites
				Photosynthesis-antenna Proteins
				Porphrin and Chlorophyll Metabolism
				Proteosome
				mRNA Survillence Pathway

The current study represents a novel framework to unravel big data analytics in terms of transcriptomics, genomics, proteomics, metabolomics, and phenomics. Additionally, this framework can be employed to decipher the regulatory mechanism by controlling elements such as miRNA, transcription factor, and cis-regulatory elements. This framework can also be used for conducting differential and comparative analysis for multi-datasets and drug-target identification. To demonstrate one of the applications of this framework, the regulatory role of miRNA targets within healthy and diseased conditions in *J.curcas* is used as a sample to understand big data analytics in terms of RNA-seq transcriptomics. This was achieved by constructing a bipartite network, followed by correlation analysis, GO inferred network, and finally co-expression network, to show that comparative analysis can help in identifying regulatory genes in JH and JV. The proposed framework may help experimental and computational biologists to analyse their own data and infer meaningful biological information.

## Conclusions

Through various plant miRNA identification and network construction techniques, a remarkable amount of information has been obtained, facilitating the construction of several biological networks. However, identifying intra-network nodes that cause variations in phenotypes remain a major challenge. With the application of transcriptome-wide strategies to elucidate biological networks in multiple sublevels, the effects on phenotypic variation can be understood. Here, we also propose future experimental validation of selected targets to confirm the regulatory roles of miRNA for predicted targets. Given the large-scale availability of transcriptome data, this framework can aid in comparative analysis to decipher the key driver nodes considerably affecting a phenotype.

## Methods

### Data Assortment

Two to three healthy and symptomatic virus-infected younger apical leaves were collected from different mature plants of the *J. curcas* genotype IC561235 from the experimental farm of Himalayan Forest Research Institute at Jwalaji, Himachal Pradesh, India. The raw data were generated using NextSeq. The raw reads were filtered using Trimmomatic (v 0.30) with a quality value of >20, and other contaminants, such as adapters, were also trimmed. The reference genome of *J. curcas* was downloaded from the Jatropha Genome Database (http://www.kazusa.or.jp/jatropha/). The Illumina NextSeq transcriptome data for both samples were separately mapped to the Jatropha reference genome by using BWA, version 0.7.5a (http://bio-bwa. sourceforge.net/), with default settings. The software package SAMtools (http://samtools.sourceforge.net/) was used to convert the sequence alignment/mapfile to the sorted binary alignment/map (BAM) file. The mapped reads ratio for the reference in each dataset was calculated by applying the flagstat command of SAMtools software to the BAM file. Differential raw reads from JH and JV leaf transcriptomes were retrieved from the publicly accessible repository Next Generation Sequencing and Analysis Resources http://14.139.240.55/download.php31.

### miRNA Identification

The annotation of high-quality reads was performed by comparing them against the non redundant database downloaded from the National Centre for Biotechnology Information, followed by the quantification of high-quality reads from JH and JV transcriptomes. miRNA identification was performed using in-house Perl scripts by using Zhang *et al*.’s algorithm^45^. The local database of mature miRNAs based on data obtained from the miRbase^46^ and plant microRNA database^47^ was constructed, and the in-house Perl script was used to identify precursor miRNAs from transcriptomes by using the parameters of sequence similarity of 100% and an e-value cutoff of 1e^−5^. After removing redundant entries, miRNAs were classified into their respective families. To predict the secondary structure, we adopted the approach of the mfold software for sequences containing not more than 4 mismatches^48^. The parameters considered for miRNA identification were as follows: 1) selection of an RNA sequence as a candidate miRNA precursor, 2) RNA sequences should fold into an appropriate stem-loop hairpin secondary structure, 3) a mature miRNA sequence site is in one arm of the hairpin structure, 4) miRNAs should have less than seven mismatches with the opposite miRNA sequence in the other arm, 5) no loop or break in miRNA sequences,and 6) predicted secondary structures should have high negative minimum fold energies (less than or equal to −20 kcal/mol)^48^.

### miRNA Target Prediction

The plant small RNA (psRNA) target (http://plantgrn.noble.org/psRNATarget/) analysis server was used to predict mRNA targets corresponding to identified miRNAs based on customised parameters^49^. Prediction analysis was performed using the option of user-submitted small RNA transcripts. The parameters considered for this analysis were as follows: 1)maximum expectation value = 3, 2) length for complementary scoring (upsize) = 20, 3) number of top target genes for each small RNA = 200, 4) target accessibility-allowed maximum energy to unpair the target site = 25, 5) flanking length around the target site for target accessibility analysis was 17 base pairs in upstream and 13 base pairs in downstream, and 6) the range of central mismatch leading to translational inhibition was 9–11 nucleotides. miRNA targets were classified into their respective miRNA families.

To cross-check psRNA-based predicted results, we performed target prediction analysis by using the TargetFinder Perl script downloaded from https://github.com/carringtonlab/TargetFinder. This script also provided the same results as those predicted using the psRNA target. Moreover, we manually compared miRNA and mRNA results by using shell scripting to evaluate the presence of false positive hits. However, all psRNA-based predictions were consistent with the results of other two methods.

### miRNA–mRNA Interaction Network Analysis

**Figure 6:**
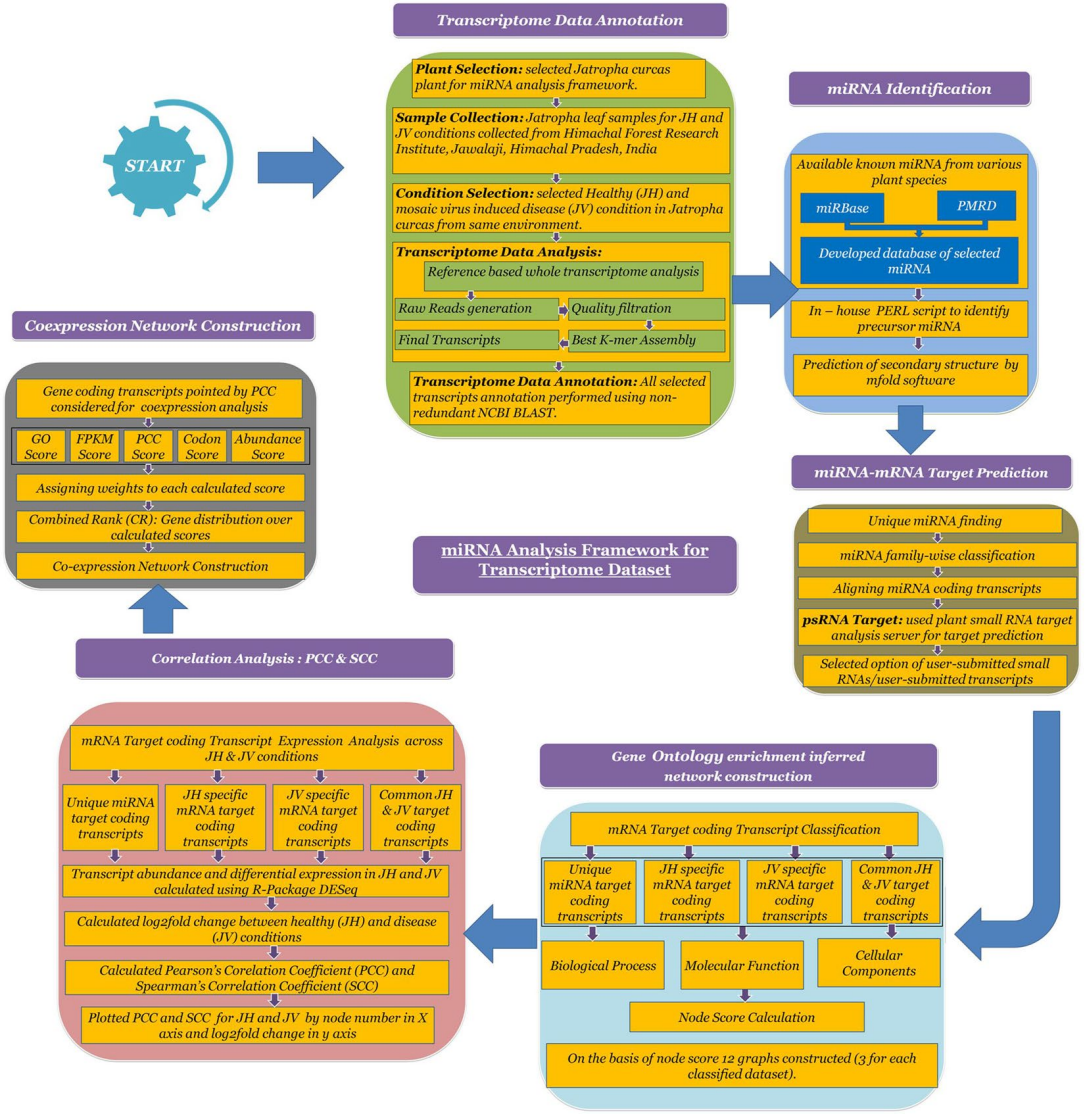
miRNA analysis framework workflow consisting 6 modules; Transcriptome Data Annotation, miRNA identification, miRNA-mRNA target prediction, gene ontology enrichment inferred network construction, correlation analysis - PCC scoring function and co-expression network construction.

Advances in network biology indicate on the fact that cellular networks are ruled by universal laws and deal with a new conceptual framework that can potentially transform our view of biology and disease pathologies^50^. The framework for network analysis is shown in Fig. 6.

### Interaction Matrix Construction

The adjacent matrix of the miRNA target network can be represented as follows:

1 $$Ai\quad j=\{\begin{array}{l}1\,if\,i \sim j\\ \,0\,otherwise\end{array}$$

where *i* represents miRNA and *j* represents the association between miRNA and its targets.

### Bipartite Network Construction

An undirected graph where G = (V, E) in which V can be partitioned into two sets, V_1_ and V_2_, such that (u, v) є E implies either u є V_1_ and v є V_2_ or v є V_1_ and u є V_2_ can be referred as a bipartite graph. In simple words, a network is called bipartite if its nodes can be divided into two groups in such a manner that nodes in one group are connected to nodes in the other group with no or sparse connexion existing within the same group. The directed bipartite network can be represented as shown in Fig. 1D. Three bipartite networks were constructed: healthy specific miRNA and its target, disease-specific miRNA and its target, and same miRNA in healthy and diseased conditions but different targets across healthy and diseased conditions. In addition, we examined the GO of selected nodes to understand the association between JH and JV.

### GO enrichment inferred network

GO analysis deals with three components, namely BPs, MFs, and CCs. BLAST2GO^51^ was used to link selected transcripts to map with the GO database in terms of BPs, MFs, and CCs. Scoring function can be defined as follows:

2 $$score(g)=\sum _{{g}_{\alpha }\in desc(g)}gp({g}_{\alpha })\cdot {\alpha }^{dist(g,{g}_{\alpha })}$$

where

- desc(g) represents all the descendant terms for a given GO term g
- dist(g, ga) represents the number of edges between the GO term g and the GO term ga
- g represents the element of GO, where GO is the whole set of all GO terms
- gp(g) represents the number of gene outcomes given to a given GO term g

The transcripts that belonged to the same category were clustered. A node score function was defined for all transcripts targeted by miRNAs in JH and JV tissues. Transcripts that had the same score were clustered in the same rectangle. Interconnection from one cluster to another cluster was performed on the basis of their respective association based on the node score.

### Degree and Correlation Analysis

The degree of a node in an undirected graph is the number of connexions or edges a node has with other nodes, and it is defined as *deg*(*i*) = *k*(*i*) = *|N*(*i*)*|* where *N*(*i*) is the number of the neighbours of node *i*. The degree distribution *p*(*k*) reveals the fraction of vertices with degree *k*. To find the correlation between constructed bipartite networks, Pearson’s correlation analysis was performed. PCC measures the linear correlation (r) between two variables.

3 $$r=\frac{[{M}^{-1}{\sum }_{i=1}^{M}\,{j}_{i}{k}_{i}]-[{M}^{-1}{\sum }_{i=1}^{M}\frac{1}{2}{({j}_{i}+{k}_{i})}^{2}]}{[{M}^{-1}{\sum }_{i=1}^{M}\frac{1}{2}({{j}_{i}}^{2}+{{k}_{i}}^{2})]-[{M}^{-1}{\sum }_{i=1}^{M}\frac{1}{2}{({j}_{i}+{k}_{i})}^{2}]}$$

where *ji* and *ki* are the degrees of targets at both the ends of the *ith* connexion, and *M* represents total connexions in the network.

A Perl script was used to calculate Pearson’s correlation value for each pair of the identical transcript in healthy and diseased conditions on the basis of FPKM values by using R package DESeq^26^. Only those transcripts that were targeted by miRNAs in both the conditions were considered for analysis and further used for co-expression network construction.

### Co-expression Network Reconstruction

**Figure 7:**
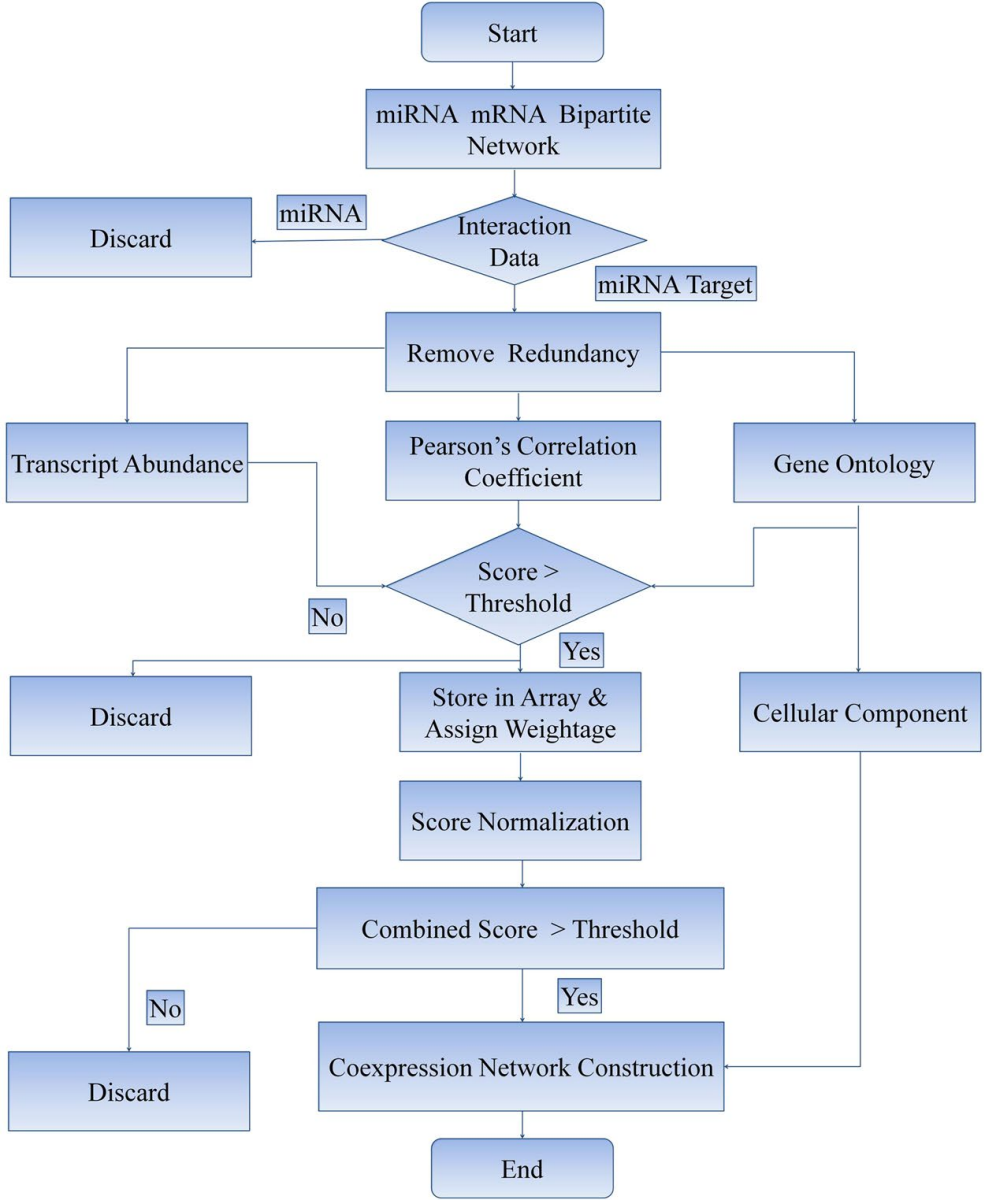
Workflow diagram for co-expression network construction.

A co-expression network is an undirected graph, with every node representing a gene or transcript and every edge representing the connection between these nodes. In this study, we used an in-house Perl script to calculate gene co-expression; we calculated various scores, assigned weights to each score, and finally generated a combined score. Out of total score i.e. 1, weights were assigned to every parameter; transcript abundance (score 0.4), GO (score = 0.3), PCC (score = 0.3). Based on combined score, we considered top 20 genes associated with query gene and plotted network in Cytoscape. Systematic workflow for co-expression analysis is shown in Fig. 7.

## Electronic supplementary material

Supplementary File 1

Supplementary File 2
