# Publisher Correction: A novel miRNA analysis framework to analyze differential biological networks

**DOI:** 10.1038/s41598-018-20355-8

**Published:** 2018-02-02

**Authors:** Ankush Bansal, Tiratha Raj Singh, Rajinder Singh Chauhan

**Affiliations:** 1 Department of Biotechnology and Bioinformatics, Jaypee University of Information Technology, Waknaghat, 173234 Solan, H.P. India; 2 Department of Biotechnology, Bennett University- A Times Group Initiative, TechZone II, Greater Noida, 201310 Uttar Pradesh India

Correction to: *Scientific Reports* 10.1038/s41598-017-14973-x, published online 03 November 2017

The original HTML version of this Article contained a typographical error in the publication date ‘03 November 2017’ which was incorrectly given as ‘06 November 2017’. This has now been corrected in the HTML version of the Article.

