# Supplementary material for: A novel miRNA analysis framework to analyze differential biological networks
Source: Sci Rep. 2017 Nov 3;7:14604. doi: 10.1038/s41598-017-14973-x (PMC5668248; doi:10.1038/s41598-017-14973-x)

A novel miRNA analysis framework to analyze differential biological networks

Ankush Bansal[1], Tiratha Raj Singh[1] & Rajinder Singh Chauhan[2]*

[1]Department of Biotechnology and Bioinformatics, Jaypee University of Information Technology, Waknaghat- 173234, Solan (H.P.), India

[2]Department of Biotechnology, Bennett University- A Times Group Initiative, TechZone II, Greater Noida- 201310, Uttar Pradesh, India

***Corresponding author**

Prof. Rajinder Singh Chauhan

Email Id: rajinder.chauhan@bennett.edu.in

Supplementary Information

Figure Legends

Supplementary Fig 1 Top ten Biological Process (BP) in Jatropha Healthy (JH) condition

Supplementary Fig 2 Top ten Molecular Functions (MF) in Jatropha Healthy (JH) condition

Supplementary Fig 3 Top ten Cellular Components (CC) in Jatropha Healthy (JH) condition

Supplementary Fig 4 Top ten Biological Processes (BP) in Jatropha virus infected (JV) condition

Supplementary Fig 5 Top ten Molecular Functions (MF) in Jatropha virus infected (JV) condition

Supplementary Fig 6 Top ten Cellular Components (CC) in Jatropha virus infected (JV) condition

Supplementary Fig 7 miRNA target co-expression and score based network reconstruction with associated pathways; selected miRNA are shown in first layer while co-expressed transcripts from transcriptome, shortlisted candidate targets based on the score and corresponding pathways are represented in second, third and fourth layer respectively

Supplementary Fig 8 miRNA target co-expression and score based network reconstruction for miR-5021; selected miRNA are shown in first layer while co-expressed transcripts from transcriptome, shortlisted candidate targets based on the score and corresponding pathways are represented in second, third and fourth layer respectively

Supplementary Fig 1 Top ten Biological Process (BP) in Jatropha Healthy (JH) condition

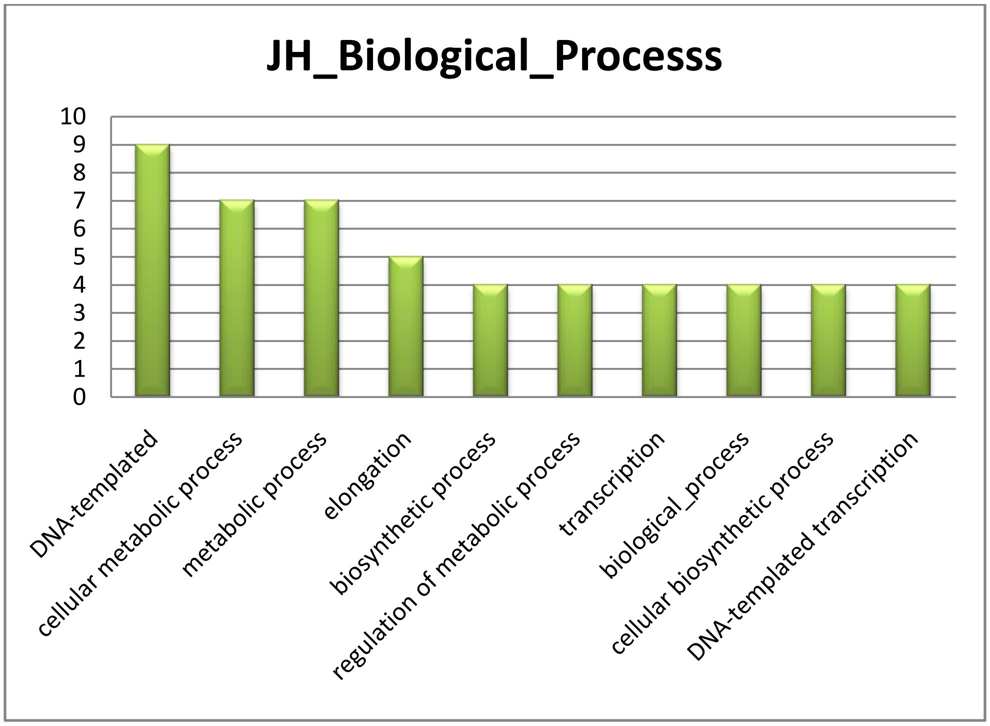

Supplementary Fig 2 Top ten Molecular Functions (MF) in Jatropha Healthy (JH) condition

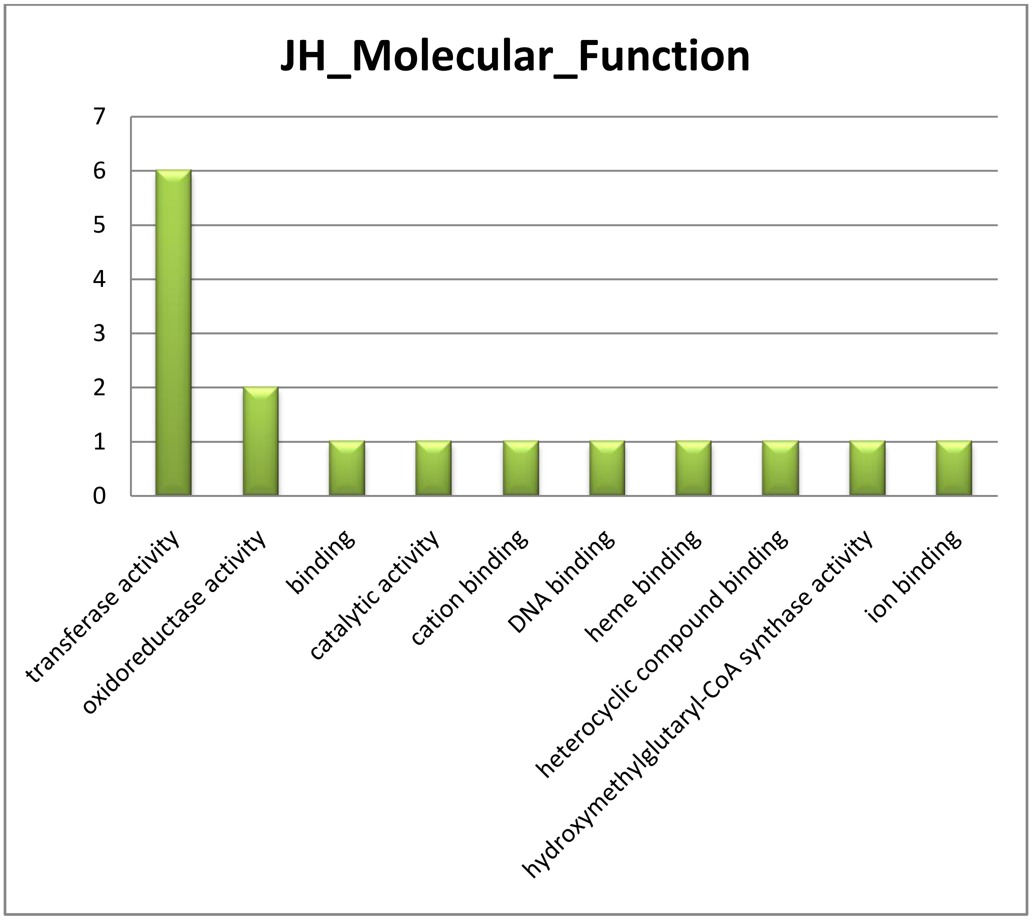

Supplementary Fig 3 Top ten Cellular Components (CC) in Jatropha Healthy (JH) condition

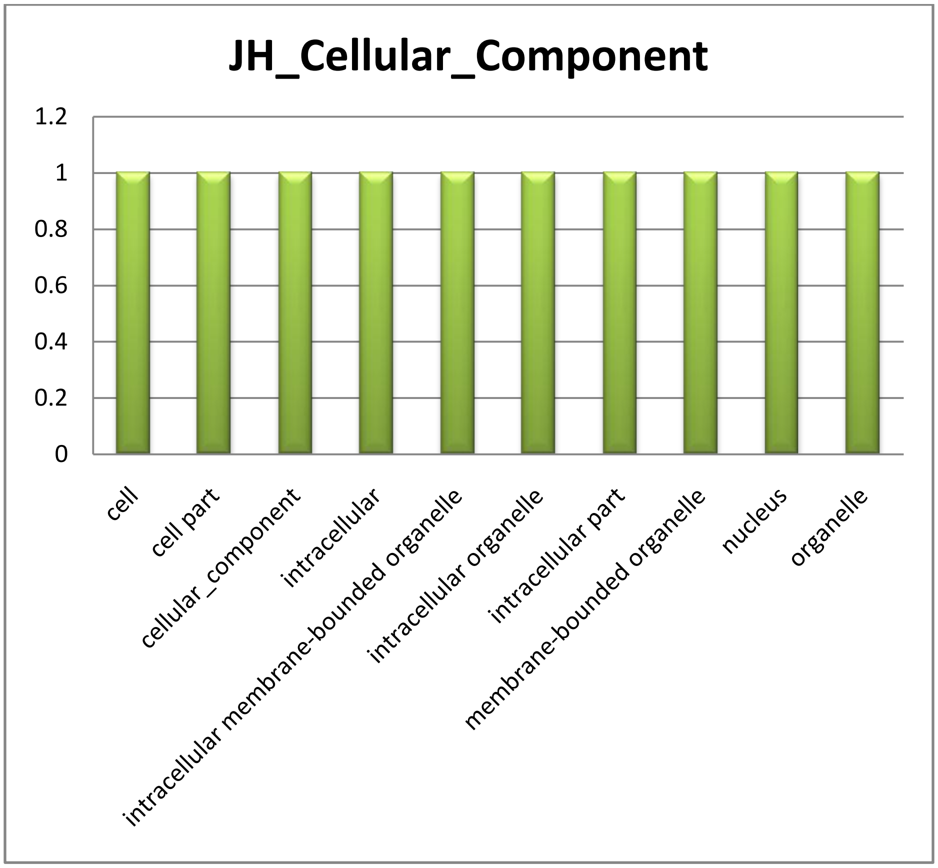

Supplementary Fig 4 Top ten Biological Processes (BP) in Jatropha virus infected (JV) condition

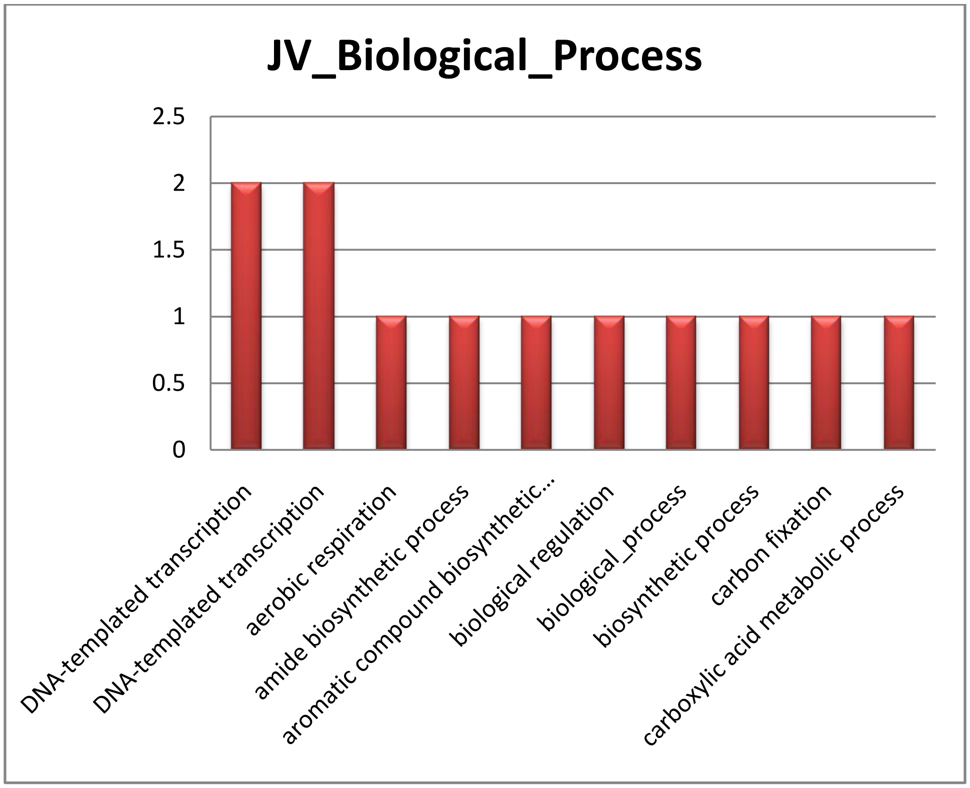

Supplementary Fig 5 Top ten Molecular Functions (MF) in Jatropha virus infected (JV) condition

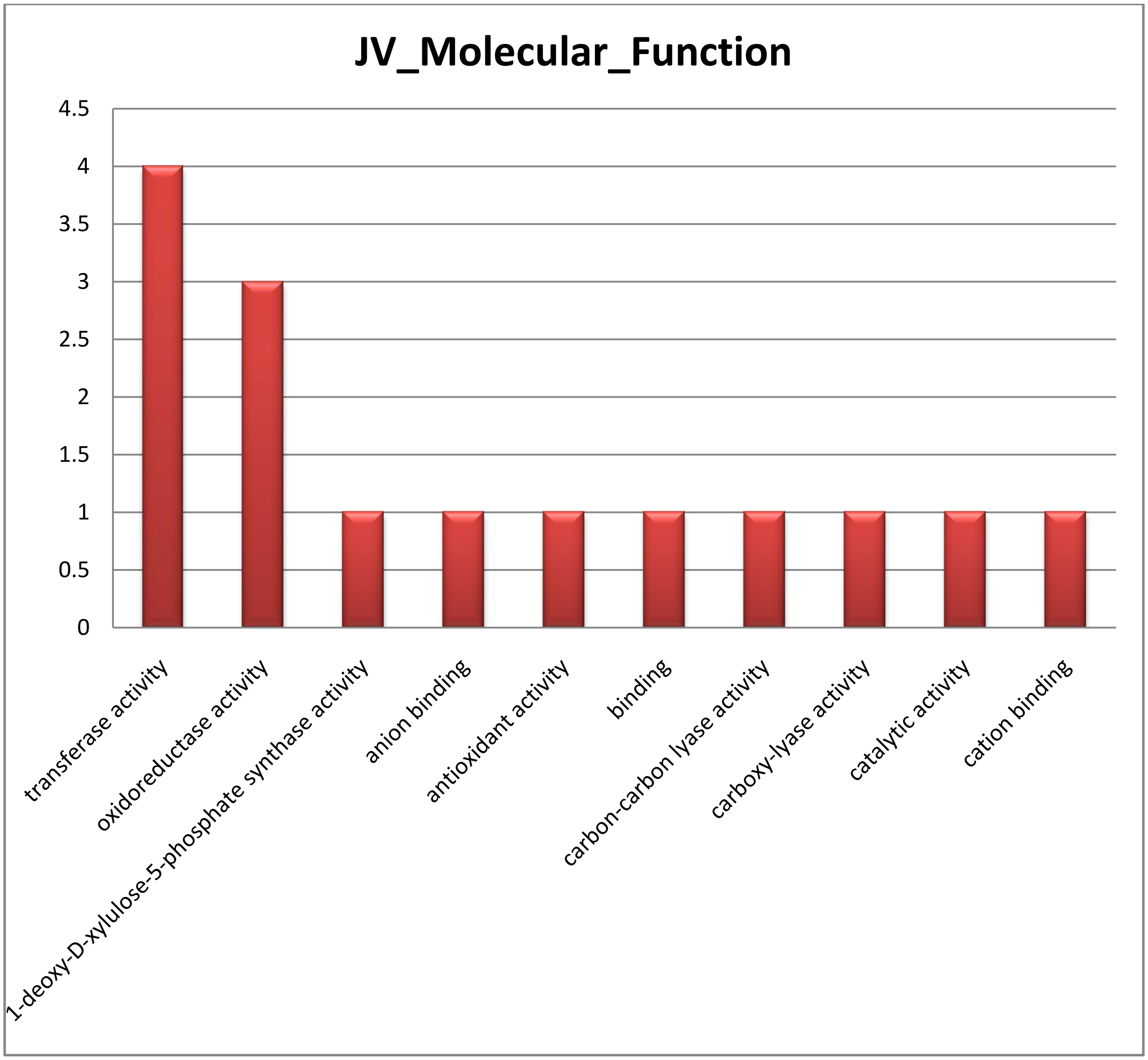

Supplementary Fig 6 Top ten Cellular Components (CC) in Jatropha virus infected (JV) condition

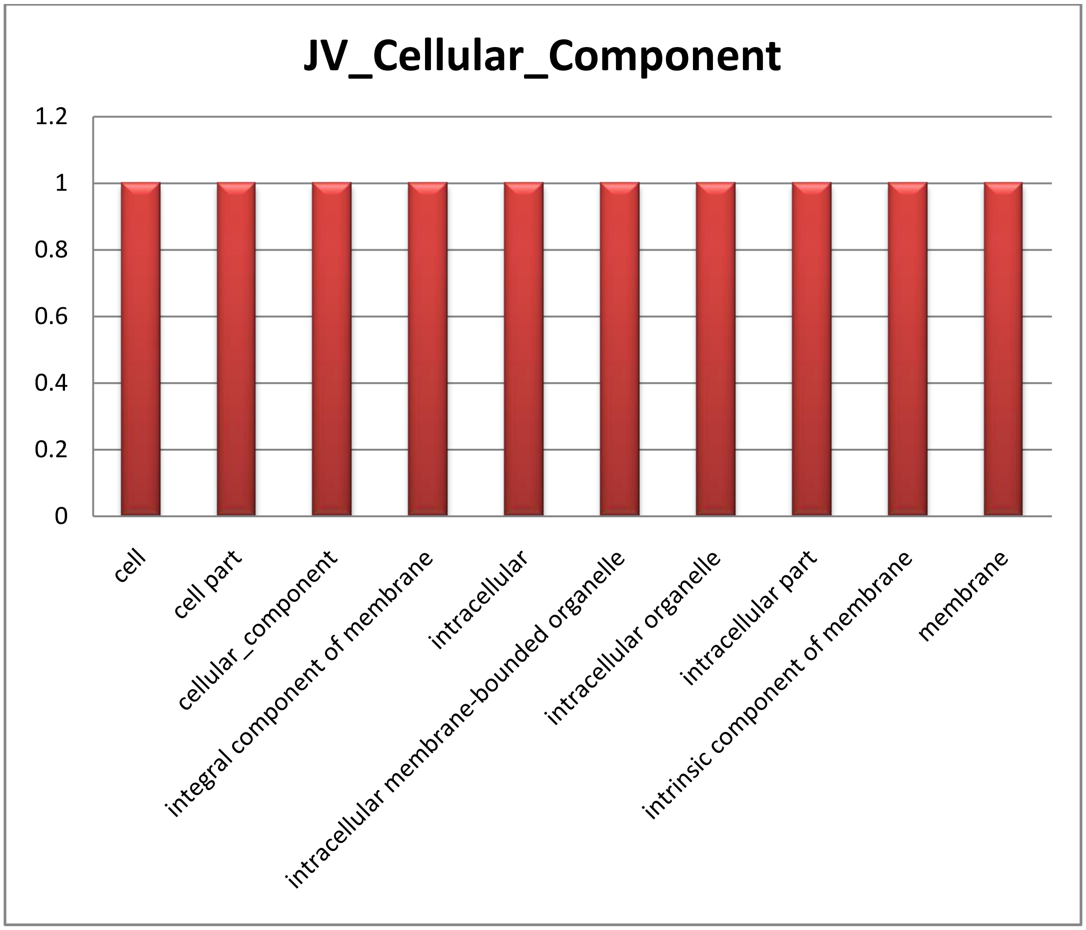

Supplementary Fig 7 miRNA target co-expression and score based network reconstruction with associated pathways; selected miRNA are shown in first layer while co-expressed transcripts from transcriptome, shortlisted candidate targets based on the score and corresponding pathways are represented in second, third and fourth layer respectively

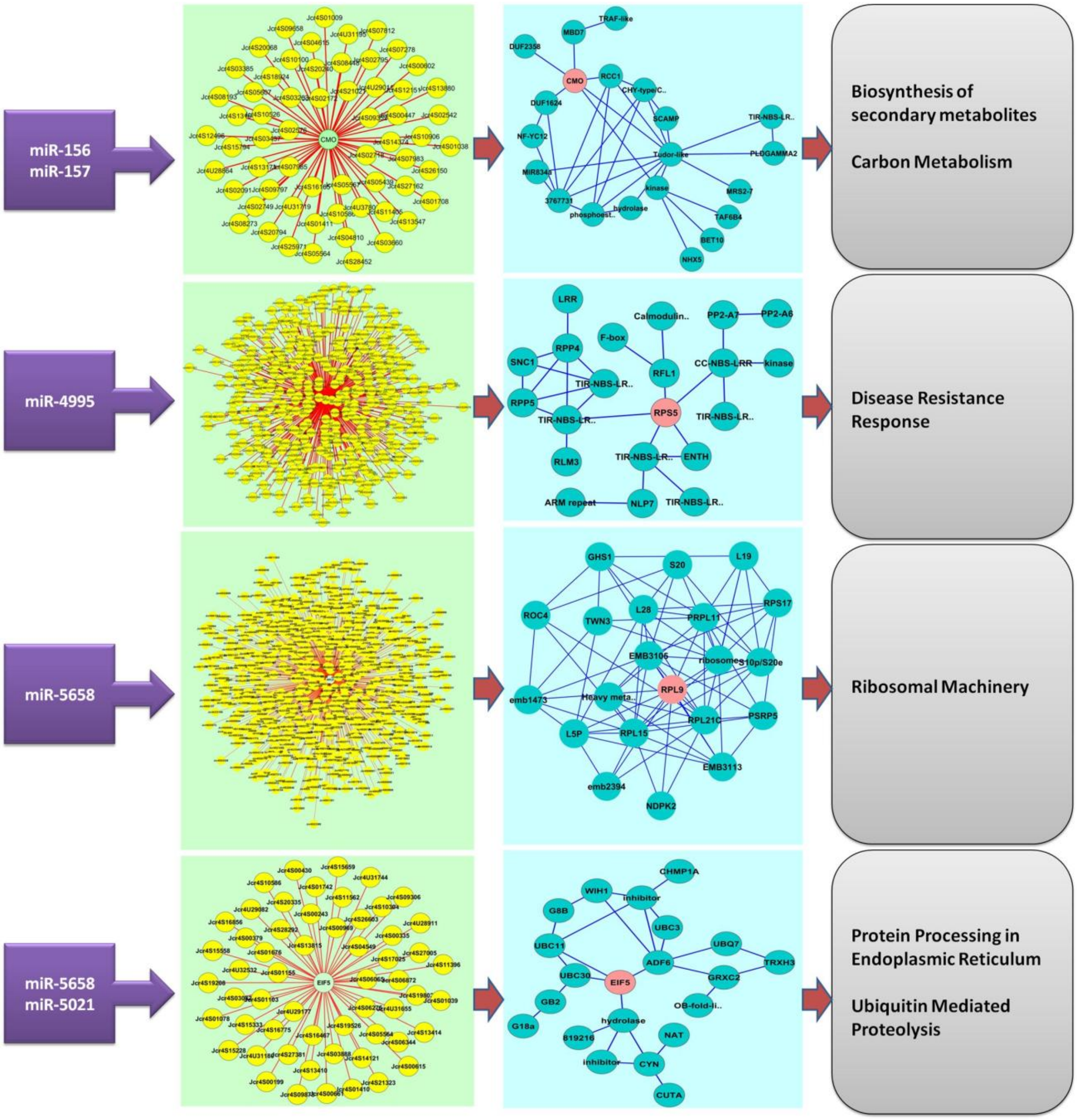

Supplementary Fig 8 miRNA target co-expression and score based network reconstruction for miR-5021; selected miRNA are shown in first layer while co-expressed transcripts from transcriptome, shortlisted candidate targets based on the score and corresponding pathways are represented in second, third and fourth layer respectively

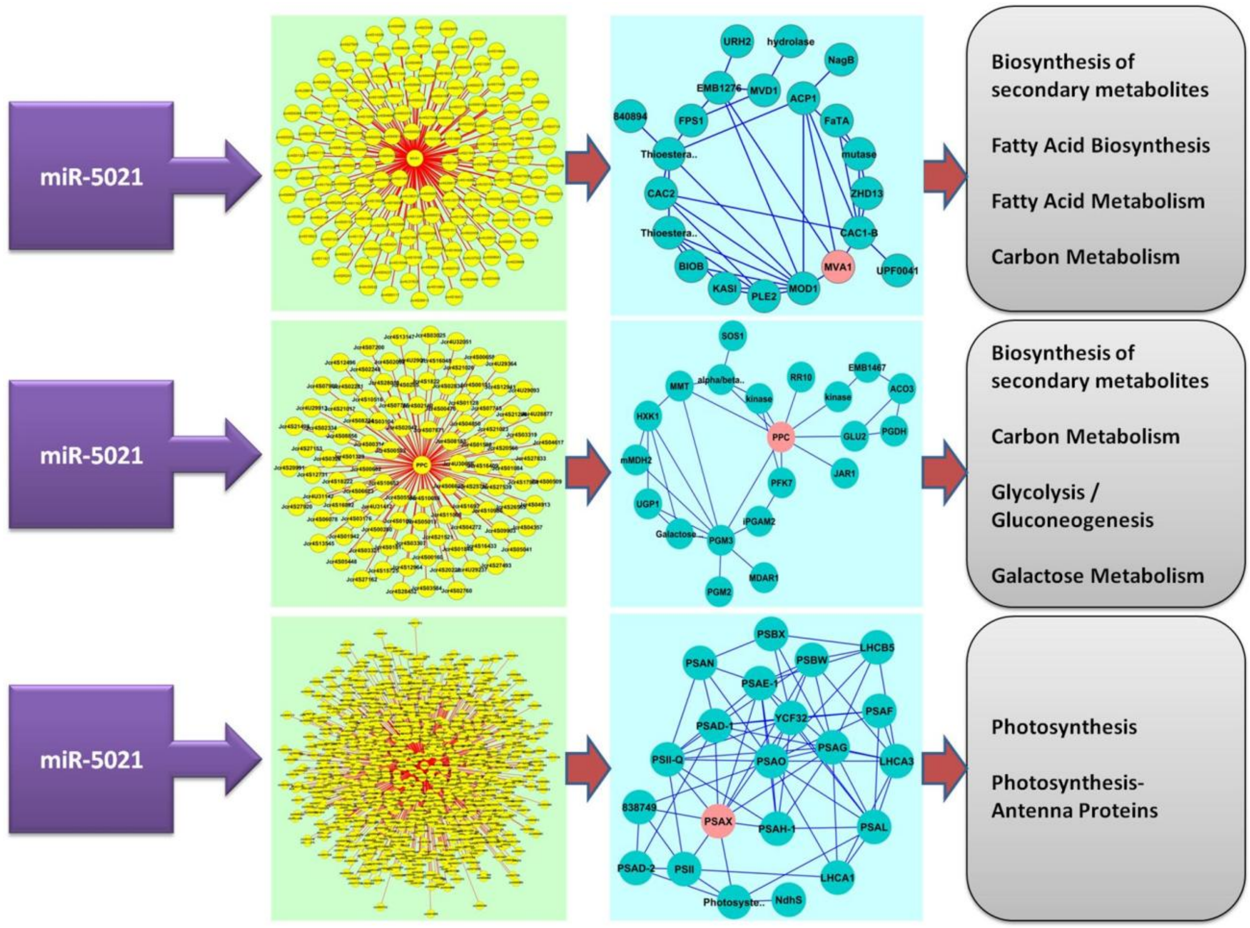

Supplement: Supplementary file 1 [file 41598_2017_14973_MOESM1_ESM.pdf]
